# Ikaite nucleation at 35 °C challenges the use of glendonite as a paleotemperature indicator

**DOI:** 10.1038/s41598-020-64751-5

**Published:** 2020-05-18

**Authors:** Elin Tollefsen, Tonci Balic-Zunic, Carl-Magnus Mörth, Volker Brüchert, Cheng Choo Lee, Alasdair Skelton

**Affiliations:** 1 Department of Geological Sciences, Stockholm University, 106 91 Stockholm, Sweden; 2 Department of Geosciences and Natural Resource Management, University of Copenhagen, 1350 Copenhagen, Denmark; 3 Chemical Biological Centre, Umeå University, 901 87 Umeå, Sweden

**Keywords:** Mineralogy, Geochemistry, Palaeoclimate

## Abstract

Glendonites have been found worldwide in marine sediments from the Neoproterozoic Era to the Quaternary Period. The precursor of glendonite, ikaite (CaCO_3_ · 6H_2_O), is metastable and has only been observed in nature at temperatures <7 °C. Therefore, glendonites in the sedimentary record are commonly used as paleotemperature indicators. However, several laboratory experiments have shown that the mineral can nucleate at temperatures>7 °C. Here we investigate the nucleation range for ikaite as a function of temperature and pH. We found that ikaite precipitated at temperatures of at least 35 °C at pH 9.3 −10.3 from a mixture of natural seawater and sodium carbonate rich solution. At pH 9.3, we observed pseudomorphic replacement of ikaite by porous calcite during the duration of the experiment (c. 5 hours). These results imply that ikaite can form at relatively high temperatures but will then be rapidly replaced by a calcite pseudomorph. This finding challenges the use of glendonites as paleotemperature indicators.

## Introduction

Calcium carbonate, one of the most common naturally occurring minerals, has an important role in the global carbon cycle. Calcite is the stable form of calcium carbonate at the Earth’s surface. However, growth of calcite is often inhibited by a range of external factors^1–3^. This explains why metastable hydrous and anhydrous carbonate minerals tend to form instead of calcite. A common example is the precipitation of aragonite instead of calcite in the oceans. The Mg/Ca ratio of the seawater controls this precipitation: when this ratio exceeds 2, aragonite is favoured because Mg acts as an inhibitor of calcite growth^1,4^.

Two hydrous forms of calcium carbonate occur instead of calcite under certain conditions: monohydrocalcite (CaCO_3_·H_2_O) and ikaite (CaCO_3_·6H_2_O). Monohydrocalcite (MHC) has been found as calcareous incrustations and the main form of calcium carbonate in Lake Issyk-Kul, Republic of Kyrgyzstan^5^ and as beach rocks around two salt lakes^6^. Experimental results and investigation of natural samples indicate that the formation of MHC requires high Mg/Ca ratios and a pH >8 in the solution from which it forms^6–8^.

The second hydrous form, ikaite, is more common than MHC despite the narrow temperature range of its stability. The mineral was first discovered as tufa columns in Ikka Fjord, SW Greenland^9,10^. Ikaite has only been observed in nature at temperatures between −2 and 7 °C^11^. At temperatures >7 °C, ikaite transforms to calcite and water pseudomorphically or by decomposition. In spite of the narrow temperature range of its stability, ikaite has been widely reported: in organic rich marine sediments^12–14^, in sea ice^15,16^, in speleothems^17^, as seasonal tufa columns in alkaline lakes^18^, as precipitates in sediments or on the shores of alkaline springs or lakes^19,20^, and as precipitates in riverbeds caused by anthropogenic pollution^21^.

In 1982, Suess *et al*. discovered authigenic ikaite crystals in marine sediments, and the resemblance of these crystals to the pseudomorph glendonite established ikaite as its precursor^12^. Structural investigation of ikaite and glendonite further confirmed this relationship^22^. Glendonites have been found worldwide in sediments (from shallow and deep marine, as well as terrestrial) of different ages (Neoproterozoic to Quaternary). Examples include Permo-Carboniferous marine sediments in Sydney Basin, Australia^23^, marine shales in the Marine Dwyka beds, South Africa^24^, Jurassic and Lower Cretaceous marine sediments in Northern Siberia^25^, deep and shallow marine sediments in Spitsbergen^26^, and organic rich shallow marine sediments in Northern Germany^27^. Glendonites can be large and have been reported up to >1 m in length^28^.

The narrow temperature range of ikaite formation observed in nature has motivated using the mineral and its pseudomorphs as an indicator of paleotemperature conditions^29^. Glendonites have also been suggested as an indicator of extreme low temperature metamorphism in Neoproterozoic sediments^30^. However, in addition to cold temperatures, ikaite precipitation requires high alkalinity^31^, high pH^32^ and the inhibition of calcite growth which has been argued to occur due to the presence of phosphate^31^ or magnesium^33^. Moreover, in laboratory experiments ikaite has been reported to precipitate at a wider range of temperature than seen in natural environments. Clarkson *et al*. (1992) precipitated ikaite from supersaturated solutions at 15 °C with triphosphate as an inhibitor of calcite nucleation^34^. Stockmann *et al*. (2018) also precipitated ikaite at 15 °C but without phosphate^35^. In another set of experiments, magnesium from natural seawater inhibited nucleation of calcite in favour of ikaite^33^. The results from these experiments may suggest that ikaite nucleation can also occur at temperature >7 °C in the natural environment. A recent study by Popov *et al*. (2019) of fossil records in glendonite bearing strata suggested that ikaite nucleation probably occurred at water temperature >40 °C^36^. These findings call into question the use of ikaite as a paleotemperature indicator. In this study, we address this issue with a series of experiments on ikaite nucleation as a function of temperature and pH.

## Methods

### Experimental setup and material

In total 39 experiments were performed at different temperatures and pH to investigate the precipitation of ikaite. The experimental setup was similar to the methods described by Stockmann *et al*. (2018) and Tollefsen *et al*. (2018) with the following modifications^33,35^:

The samples were precipitated from a 50–50 mixture of two solutions: Solution 1) was surface seawater collected nearby the island of Andøya, Norway (Table 1). Solution 2) was prepared by mixing 0.1 M Na_2_CO_3_ and 0.1 M NaHCO_3_ at different proportion, 1:5, 1:3, 1:2, 1:1 and 3:1, which represent solutions with pH 9.49, 9.70, 9.86, 10.14 and 10.59 at 5 °C respectively.

**Table 1:** Chemical composition of seawater measured by ion-coupled plasma mass spectrometry.

Component	Andøya seawater
	N 69°16.981′ E 15°52.462'
conductivity (mS/cm)	50.5
salinity (‰)	33.1
pH	8.07
temp (°C)	5
Alkalinity mmol/L	2.1378 ± 0.002
Na^+^ mmol/L	432.15 ± 1.18
K^+^ mmol/L	9.60 ± 1.06
Ca^2+^ mmol/L	8.83 ± 0.22
Mg^2+^ mmol/L	45.60 ± 0.50
Sr^2+^ mmol/L	0.08 ± 0.49
Cl^−^ mmol/L	533.59 ± 2.37
SO_4_^2−^ mmol/L	27.31 ± 0.50
Br^−^ mmol/L	n.d.
PO_4_^3−^ μmol/L	<0.016
Total C mmol/L	2.10 ± 0.04

Note: n.d. not detected.

The seawater was filtered through Munktell Qualitative Filter Paper grade 3 before use and stored in a cooling room at 5 °C. This removes any particulate matter >10 µm in size. We consider it unlikely that finer particulate matter, if present, affected the outcome of our experiments. This is because Tollefsen *et al*. (2018) used the same approach and obtained comparable results for synthetic seawater and natural seawater from the same location. Solution 2 was prepared using Na_2_CO_3_ and NaHCO_3_ powders from Merck dissolved in 1 litre of ultrapure deionized water (MilliQ resistivity >18.2 MΩ cm).

The two solutions and their mixtures were kept in water baths during the experiments to maintain the desired temperature. We ran experiments at temperatures from 5 to 35 °C. The mixing rate was 0.67 ± 0.21 ml/min and when ~300 ml of mixture was obtained, the experiment was stopped. The resulting mixture was put in a cooling room (5 °C) overnight. The next day, the mixture was filtered through Munktell Qualitative Filter Paper 00 K and kept overnight to dry in the cooling room. Thereafter, the precipitate was collected, weighed and stored in a freezer (−18 °C).

### XRD analysis

Precipitated minerals were identified by X-ray powder diffraction (XRD) using an X Pert Pro instrument from PANalytical and the program Data Colllector at the Swedish Museum of Natural History. The sample holder was kept in a freezer (−18 °C) for 10 min before analysis to preserve the ikaite crystals. The measurement program used was Absolute Scan 5–70°2θ with a runtime of 11 min. The program Topas version 6 (Bruker AXS product) was used to identify and quantify the different phases in the samples by Rietveld refinement. In 7 samples, amorphous calcium carbonate (ACC) was present, which was quantified by Rietveld refinement of the XRD data after addition of a quartz standard to the sample in weighed proportions. Distinguishing calcite from Mg-calcite was based on unit cell data obtained from the Rietveld refinement (Supplementary Fig. S1).

### Cryo-SEM imaging

Scanning electron microscope (SEM) was used to visualise 19 of 39 samples. The instrument used was a Carl Zeiss Merlin field-emission cryogenic scanning electron microscope, fitted with a Quorum Technologies PP3000T cryo-preparation system at Umeå University. The frozen precipitate was glued onto a support and placed into liquid nitrogen slush before transferring to the instrument chamber. The images were taken at temperature c. −140 °C using in-chamber secondary electron detector (ETD) at an accelerating voltage of 2 kV.

### PhreeqC

We used the program PhreeqC Interactive^37^ version 3.4.0 for geochemical modelling of all the experiments to calculate values for pH and ionic speciation. Saturation indices (SI) for the carbonate minerals were taken from the PhreeqC llnl database, except for ikaite (CaCO_3_·6H_2_O), vaterite (CaCO_3_) and amorphous calcium carbonate (CaCO_3_·H_2_O). These data were added separately, using solubility constants from Bischoff *et al*. (1993) for ikaite^31^, Plummer and Busenberg (1982) for vaterite^38^ and Brecevic and Nielsen (1989) for ACC^39^. The SI was defined as follows: SI = log (IAP/Ksp), with IAP = ion activity product and Ksp = solubility constant.

### ICP and IC20 analyses

We dissolved and diluted 27 of 39 samples for cation analysis with an Inductively coupled plasma - atomic emission spectroscopy (ICP-OES, Thermo ICAP 6500) with autosampler ASX520 from CETAC and using a spray nebulizer. The seawater sample from Andøya was also analysed with the same instrument. The concentrations of anions in the seawater were measured with an IC20 Ion Chromatograph from Dionex.

## Results

From the experiments, we obtained 39 samples precipitated from by mixing natural seawater (Table 1) with a solution containing 0.1 M of Na_2_CO_3_ and 0.1 M of NaHCO_3_ in different proportions (1:5, 1:3, 1:2, 1:1 and 3:1). The calculated pH of the parent solutions ranged from 9.09 to 10.31 and calculated alkalinities ranged from 59 to 89 meq/kg (PhreeqC).

### Mineral identification

Based on XRD analysis, we identified seven minerals (Supplementary Fig. S2): calcite (CaCO_3_), Mg-calcite ((Ca,Mg)CO_3_), aragonite (CaCO_3_), monohydrocalcite (CaCO_3_·H_2_O), ikaite (CaCO_3_·6H_2_O), nesquehonite (MgCO_3_·3H_2_O) and one amorphous phase: Mg rich amorphous calcium carbonate (Ca_(1−x)_Mg_x_CO_3_·H_2_O). We estimated the amount of Mg in the amorphous calcium carbonate (ACC) to be 35 ± 10 mol. % from ICP-AES measurements and measured proportions of ACC, ikaite (which can only incorporate traces of Mg) and nesquehonite in the sample. The refinement of Mg occupancy for the Mg-calcite was calculated to be ~10 mol. %. This is within the range observed in marine sediments and Mg-calcite cements^40^.

### Experimental results

The experiments were designed to test for ikaite nucleation at temperatures between 5 and 35 °C at different pH. We organised the experiments into five series, each with a different pH: Series 1 (pH 9.09; exp. 1–4), series 2 (pH 9.32; exp. 5–11), series 3 (pH 9.50; exp. 12–18), series 4 (pH 9.82; exp. 19–25) and series 5 (pH 10.31; exp. 26–32). For simplicity, pH values reported in this study are those calculated at 5 °C using PhreeqC. In total, we ran 32 experiments and 7 duplicates (Table 2).

**Table 2:** X-ray diffraction (XRD) results.

Exp. nr	Calculated pH (PhreeqC) (5 °C)	Ratio Na_2_CO_3_: NaHCO_3_	T during exp. °C	Measured pH at T	T (°C)	XRD results
**1st Series**						
1	9.09	1:5	5	8.95	8.6	Mg-calcite* 75% aragonite 25%
2	9.09		15	8.76	16.7	Mg-calcite 46% aragonite 46% MHC 8%
3	9.09		25	8.71	23.6	Mg-calcite 51% aragonite 45% MHC 4%
4	9.09		35	8.62	30.9	aragonite 53% Mg-calcite 43% MHC 4%
**2nd Series**						
5	9.32	1:3	5	9.12	9.4	calcite 61% Mg-calcite 39%
6a	9.32		10	9.20	13.0	MHC 100%
6b	9.32		10	9.15	14.1	calcite 60% Mg-calcite 25% ikaite 10% MHC 5%
7a	9.32		15	9.00	16.6	calcite 90% ikaite 10%
7b	9.32		15	9.01	16.9	ikaite 74% Mg-calcite 24% calcite 2%
8a	9.32		20	9.03	20.0	calcite 80% Mg-caclite 16% ikaite 4%
8b	9.32		20	8.96	20.1	calcite 50% MHC 36% Mg-calcite 14%
9	9.32		25	8.94	23.5	MHC 93% Mg-calcite 7%
10	9.32		30	8.91	27.2	ikaite 86% MHC 8% Mg-calcite 4% calcite 2%
11	9.32		35	8.87	31.1	ikaite 67% calcite 23% MHC 8% Mg-calcite 2%
**3rd Series**						
12a	9.50	1:2	5	9.34	9.9	ikaite 100%
12b	9.50		5	9.44	9.9	ikaite 100%
12c	9.50		5	9.43	10.3	ikaite 98% calcite 2%
13	9.50		10	9.38	13.5	ikaite 100%
14	9.50		15	9.36	17.0	ikaite 100%
15	9.50		20	9.30	19.9	ikaite 100%
16	9.50		25	9.16	23.3	ikaite 97% calcite 3%
17	9.50		30	9.07	26.8	ikaite 96% calcite 4%
18	9.50		35	9.03	28.1	ikaite 87% calcite 13%
**4th Series**						
19a	9.82	1:1	5	n.m.	n.m.	ikaite 97% calcite 3% **
19b	9.82		5	9.64	9.6	ikaite 100% **
19c	9.82		5	9.64	9.3	ikaite 86% calcite 14%
20	9.82		10	9.69	13.5	ikaite 100%
21	9.82		15	9.68	17.5	ikaite 100%
22	9.82		20	9.61	20.5	ikaite 100%
23	9.82		25	9.63	23.7	ikaite 100%
24	9.82		30	9.55	25.3	ikaite 100%
25	9.82		35	9.53	29.6	ikaite 20% ACC 80%
**5th Series**						
26	10.31	3:1	5	10.11	9.5	ikaite 100%
27	10.31		10	10.20	14.0	ikaite 61% ACC 39%
28	10.31		15	10.10	16.9	ikaite 57% ACC 38% nesquehonite 5%
29	10.31		20	10.08	20.7	ikaite 25% ACC 72% nesquehonite 3% (trace calcite)
30	10.31		25	10.03	23.6	ikaite 14% ACC 83% nesquehonite 3% (trace calcite)
31	10.31		30	9.97	27.0	ikaite 16% ACC 80% nesquehonite 4% (trace calcite)
32	10.31		35	9.88	29.4	ikaite 4% ACC 94% (trace nesquehonite, halite, calcite)

Note: MHC monohydrocalcite, ACC amorphous calcium carbonate, n.m. not measured.

*Mg-calcite has been identified from XRD data (unite cell volume, a and c-axis).

**XRD results from HighScore plus.

Ikaite did not precipitate at any temperature in series 1. Instead Mg-calcite precipitated together with aragonite or together with aragonite and monohydrocalcite (MHC) (Table 2). Ikaite precipitated in most of the series 2 experiments, either as the main phase (at 15 °C, 30 °C and 35 °C) or subsidiary to MHC and/or calcite (Table 2). In a few series 2 experiments (at 5 °C and 25 °C), ikaite precipitates were not observed (Table 2).

Ikaite precipitated in all experiments in series 3, 4 and 5. In series 3 pure ikaite precipitated at all temperatures up to 20 °C in all but one experiment in which calcite also precipitated, and together with calcite at temperatures of 25 °C and higher (Table 2). In series 4 pure ikaite precipitated at all temperatures up to 30 °C in all but two experiments in which calcite also precipitated. In the experiment run at 35 °C (exp. 25) the precipitate was 80% amorphous calcium carbonate (ACC) and 20% ikaite (Table 2). In series 5 pure ikaite precipitated at 5 °C, and thereafter together with ACC at 10 °C and together with ACC and nesquehonite from 15 to 35 °C. The proportion of ACC increased with temperature (Table 2).

### Imaging by Cryo-SEM

**Figure 1:**
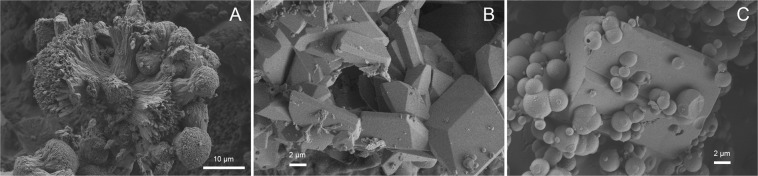
SEM images of (**A**) exp. 2 from series 1 shows small semi-spherical structures of probable Mg-calcite and fibres of probable aragonite, (**B**) exp. 17 from series 3 with euhedral ikaite crystals, and (**C**) exp. 25 from series 4 ikaite crystal partly hidden by spherical structures which could be Mg-rich ACC or pseudomorphs after vaterite.

The sample from series 1 contained spherical Mg-calcite and fibrous aragonite (Fig. 1A). The samples from series 3, 4 and 5 contained euhedral ikaite crystals (Fig. 1B). One sample from series 4 (exp. 25 run at 35 °C) and one sample from series 5 (exp. 28 run 15 °C) contained euhedral ikaite crystals together with spherical ACC (Fig. 1C).

In series 2, ikaite was observed in 6 of 10 samples (exp. 6b, 7a, 7b, 8a, 10 and 11) together with Mg-calcite and/or MHC as indicated by the XRD analyses (Table 2). In samples with >4% calcite, we observed perfectly shaped pseudomorphs after ikaite, but no typical calcite crystals (Fig. 3). Pseudomorphs after ikaite were observed in samples from experiments run at 5, 10, 15, 20 and 35 °C in series 2 and in the sample from one experiment (exp. 18) from series 3 which was run at 35 °C. In these samples, all ikaite crystals (exps. 5 and 8b) or some ikaite crystals (exps. 6b, 7a, 8a, 11 and 18) were pseudomorphically replaced and showed a porous replacement texture (Fig. 3 and Fig. 4).

**Figure 2:**
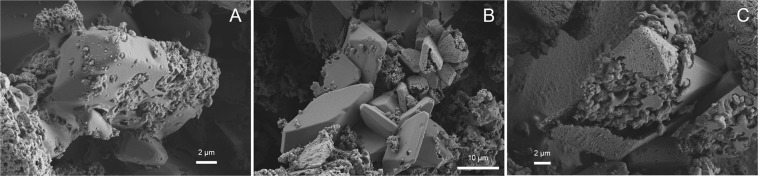
SEM images of (**A**) exp. 7b shows ikaite crystals with cavities, (**B**) exp. 11 shows euhedral ikaite crystals, pseudomorphs after ikaite and ikaite crystals with cavities, and (**C**) a single crystal from exp.11 partly replaced by calcite and with large cavities.

**Figure 3:**
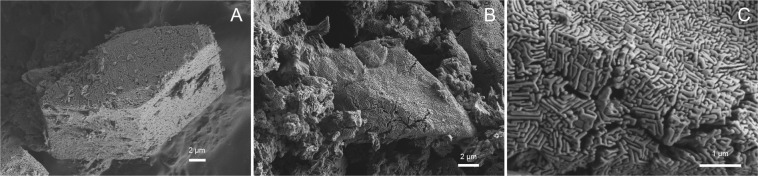
SEM images of pseudomorphs after ikaite (**A**) from exp. 8a and (**B)** from exp. 8b. (**C**) Zoomed in on the surface of the crystal in image B.

**Figure 4:**
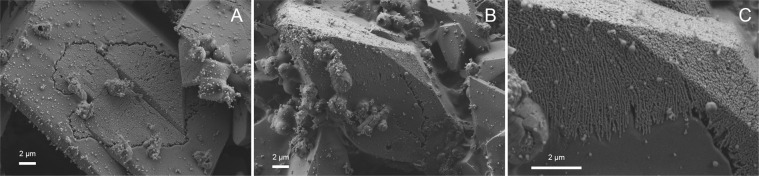
SEM image of exp. 18. Some ikaite crystals have been partly replaced in this sample. (**A**) The replacement front of ikaite occurs in the centre of the crystal towards the edges. In (**B**,**C**) the replacement front occurs at the edge of the crystal towards the centre.

In three of the samples from series 2, we did not observe pseudomorphs after ikaite. In the first sample (exp. 9 run at 25 °C), we observed large spherical MHC and spherical Mg-calcite. In the second sample (exp. 10 run at 30 °C), the main phase was ikaite which occurred as euhedral crystals. In the third sample (exp. 7b run at 15 °C), ikaite and Mg-calcite were the main phases observed. Some of the ikaite crystals in this sample had cavities (Fig. 2A). This texture was also observed in exp. 11 run at 35 °C together with euhedral ikaite crystals and pseudomorphs after ikaite (Fig. 2B,C).

In sample 18 from series 3 (run at 35 °C), we observed ikaite but also partly replaced ikaite crystals (Fig. 4). In one crystal (Fig. 4A), replacement occurs in the centre of the ikaite crystal. In a second crystal from the same sample the replacement occurs at the edge of the crystal (Fig. 4B,C).

### Modelling

The PhreeqC software calculations for each of the experiments (Supplementary Fig. S3) showed that calcite, aragonite, ikaite, MHC and ACC were supersaturated at all temperatures. Calcite and aragonite had a SI between 2 and 3, and MHC had an SI of ~1.5. Ikaite was nearly at equilibrium at low pH and high temperature (SI = 0.04), but its SI was higher (up to 1.4) at higher pH and lower temperature. Amorphous calcium carbonate was nearly at equilibrium at low pH and low temperature (SI = 0.04), but its SI was higher (0.57) at higher pH and temperature. Nesquehonite was undersaturated except for exp. 29 to 32, which is in agreement with the occurrence of this mineral in the samples from these experiments (Table 2).

### Summary of results

The experimental results showed that at pH 9.50 and 9.82 (series 3 and 4), ikaite precipitated at temperatures between 5 °C and 35 °C, and at pH 10.31 (series 5), ikaite precipitated together with ACC at temperatures between 10 °C and 35 °C. The amount of ACC increased with temperature and at 35 °C, the sample contained 94% ACC (Table 2). At pH 9.09 (series 1), ikaite did not precipitate. Instead, Mg-calcite precipitated together with aragonite at all temperatures.

At pH 9.32 (series 2), mixtures of ikaite, MHC, calcite, and Mg-calcite precipitated and pseudomorphs after ikaite were observed on Cryo-SEM images of seven samples (exps. 5, 6b, 7a, 8a, 8b, 11 and 18). These samples were also the only samples that contained high amounts of calcite (13–90%), which suggests pseudomorphic replacement of ikaite by calcite.

Because ikaite precipitated at 35 °C in series 2–5, we infer that the upper temperature limit for ikaite nucleation exceeds 35 °C. However, because ikaite content diminished with increasing temperature in series 4 and 5, we infer that this limit is close to 35 °C for these series. On the other hand, because ikaite was the main phase at 35 °C in series 2 and 3 and because Cryo-SEM images of these samples indicate that minor calcite (23 and 13%, respectively) occurred as pseudomorphs after ikaite (Figs. 2B,C and 4), we infer that the upper temperature limit for ikaite nucleation is higher than 35 °C for these series.

## Discussion

**Figure 5:**
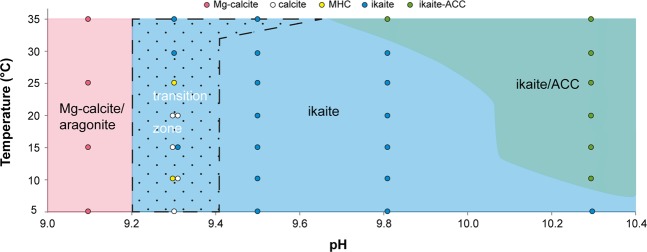
Phase diagram for calcium carbonate nucleation temperatures as a function of pH in the parent solution. In this diagram, four main zones have been defined; Mg-calcite/aragonite zone; transition zone with pseudomorphs; ikaite zone; and ikaite/ACC zone. The dots represent the results from the experiments with indication of the main phase by colour.

We constructed a stability diagram for nucleation of some anhydrous and hydrous carbonate phases as a function of pH and temperature (Fig. 5). We defined three distinct zones based on our experiments: (1) Mg-calcite/aragonite zone, (2) ikaite zone, (3) ikaite/ACC zone. Between the Mg-calcite/aragonite and ikaite fields, we define a transition zone in which several phases precipitated, and most notably, pseudomorphs after ikaite appeared within the duration of the experiments (~5 hours). In the following discussion, we will use this diagram and our experimental findings to consider the nucleation of ikaite and its pseudomorphic replacement by calcite.

### Ikaite nucleation

**Figure 6:**
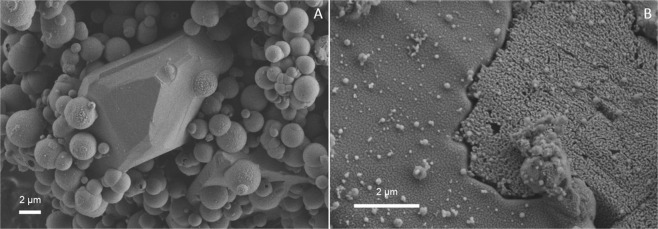
(**A**) Exp. 25 ikaite crystal closely surrounded by ACC. Some ACC spheres seem to grow out of the ikaite crystal. (**B**) Replacement textures in a ikaite crystal partly pseudomorphically replaced by calcite. A clear replacement front can be seen (exp. 18).

Ikaite has been found to occur naturally in modern settings at temperatures below 7 °C^10,12^. However, in our experiments, ikaite nucleated at much higher temperatures (up to 35 °C) in series 2–5. The upper temperature limit for ikaite nucleation was approached in series 4 (pH 9.82). In the experiment run at 35 °C, the main precipitate was ACC, which occurred as spheroids closely surrounding euhedral ikaite crystals (Fig. 6A).

Based on previous experimental work which showed that ikaite nucleation is favoured by the presence of calcite growth inhibitors such as phosphate and Mg^31,33,35^, we infer that Mg in seawater was a contributory factor which allowed for ikaite nucleation at higher temperatures in our experiments. Also, several studies have shown that the uncharged ion pair species CaCO_3_^0^ plays an important role in the multistage pathways whereby anhydrous and hydrous carbonates form^10,35,41,42^. Specifically, Gal *et al*. (1996) showed that CaCO_3_^0^ is easily hydrated and initially forms ACC and, at low temperatures, ikaite^41^. The importance of the CaCO_3_^0^ ion pair for ikaite nucleation is confirmed by our experiments: The main precipitates in series 1 (pH 9.09), which had the lowest CaCO_3_^0^ concentration in the parent solution (<1.37 mmol/kg: Supplementary Fig. S4), were Mg-calcite and aragonite together with only small amounts of MHC. In contrast, the main precipitate in series 2 (pH 9.32) at and above T = 30 °C at which the CaCO_3_^0^ concentrations approaches the Ca^2+^concentrations (1.76 and 2.22 mmol/kg respectively) in the parent solution (Supplementary Fig. S3), was ikaite (Table 2). This is despite the fact that its SI is lower than at 5 °C (Supplementary Fig. S3). The activity of the CaCO_3_^0^ ion pair is higher than the activity of the Ca^2+^ ion in all experiments (Supplementary Fig. S6). We suggest that this could highlight the importance of the CaCO_3_^0^ ion pair for ikaite nucleation as Buchardt *et al*. (2001) and Stockmann *et al*. (2018) have also previously suggested^10,35^.

### Pseudomorphic replacement of ikaite

In some of our experiments, ikaite which had precipitated at temperatures up to 35 °C, was thereafter pseudomorphically replaced by calcite within the experimental runtime of 5 hours.

Several experimental studies have investigated the breakdown of the ikaite crystal^43–45^. These studies demonstrate that the ikaite unit cell expands anisotropically with increasing temperature. The CaCO_3_^0^ ion pair in ikaite is surrounded by water molecules held together with hydrogen bonding. When ikaite is exposed to higher temperatures, the unit cell expands along the a-axis followed by the b and c-axis. This volume increase occurs just before the breakdown of the hydrogen bonds and the release of the water molecules from the ikaite structure^43,45^. In our experiments, this was observed in series 4 (pH 9.82). Here, unit cell data show a sudden increase along the a-axis and in volume at 35 °C (exp. 25; Supplementary Fig. S5). This was the only series where we found similarities in the crystal lattice data with previous studies of ikaite breakdown. One reason could be that in series 2 and 3 the temperature limit of ikaite nucleation at 35 °C with pH 9.32–9.50 was not reached despite the observation of pseudomorphs after ikaite.

In the transition zone, we observed pseudomorphs after ikaite in most samples (Table 2; Fig. 5). We speculate that the pseudomorphic replacement of ikaite is controlled not only by temperature but also by pH. A possible scenario could be that ikaite precipitation lowers the pH in the parent solution beyond the limit for metastable nucleation of ikaite and thereafter the mineral starts to transform and is replaced by calcite pseudomorphically. Calcite has been reported as the primary replacement mineral in naturally occurring pseudomorphs (glendonite) after ikaite^11,24,36,46^. Our experiments confirm this common observation. They also point to replacement of ikaite by calcite occurring at higher temperatures than previously thought.

Based on the perfect shapes of pseudomorphs, porous nature of the replacement calcite and a sharp reaction front, we propose that ikaite replacement occurs by a coupled dissolution - reprecipitation mechanism at the ikaite-calcite interface. Putnis and Putnis (2007) describe this mechanism as a coupled dissolution and nucleation process within the fluid boundary layer at the parent solid surface^47^. The generation of porosity by the reaction allows fluids and mass transport to and from the reaction interface. In this process, the original morphological structure is generally well preserved^47^. In Fig. 6B a reaction front can be seen in the crystal, with a solid surface, the interface and the generated porosity. This mechanism could explain why calcite grows in an otherwise Mg-rich solution, as follows. It has been shown that the fluid at the sharp interface can be distinct and isolated from the bulk solution^48^. The fluid at the reaction interface in our experiments is probably composed of H_2_O released by the dissolution of ikaite, and therefore calcite growth is not inhibited at the interface. We also postulate that this process took place in the solution before filtering, because otherwise we should have observed pseudomorphic replacement of ikaite in all ikaite samples. This suggests that the replacement of ikaite can occur at an early stage and at high rate if the conditions change slightly (e.g. pH and/or temperature).

**Figure 7:**
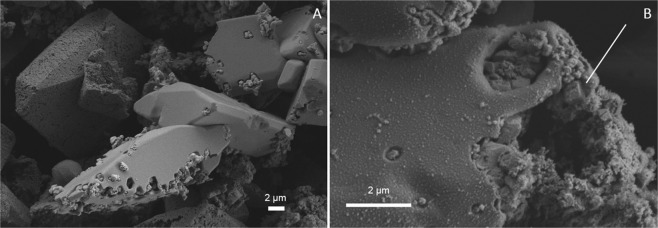
(**A**) Cryo-SEM image from exp. 11 showing euhedral ikaite crystals with small cavities and a pseudomorphically replaced ikaite crystal with porous texture in the background. (**B**) Ikaite crystal from exp. 7b with cavities filled with undefined material and small calcite crystals. The white line point to a rhombohedral calcite crystal.

**Figure 8:**
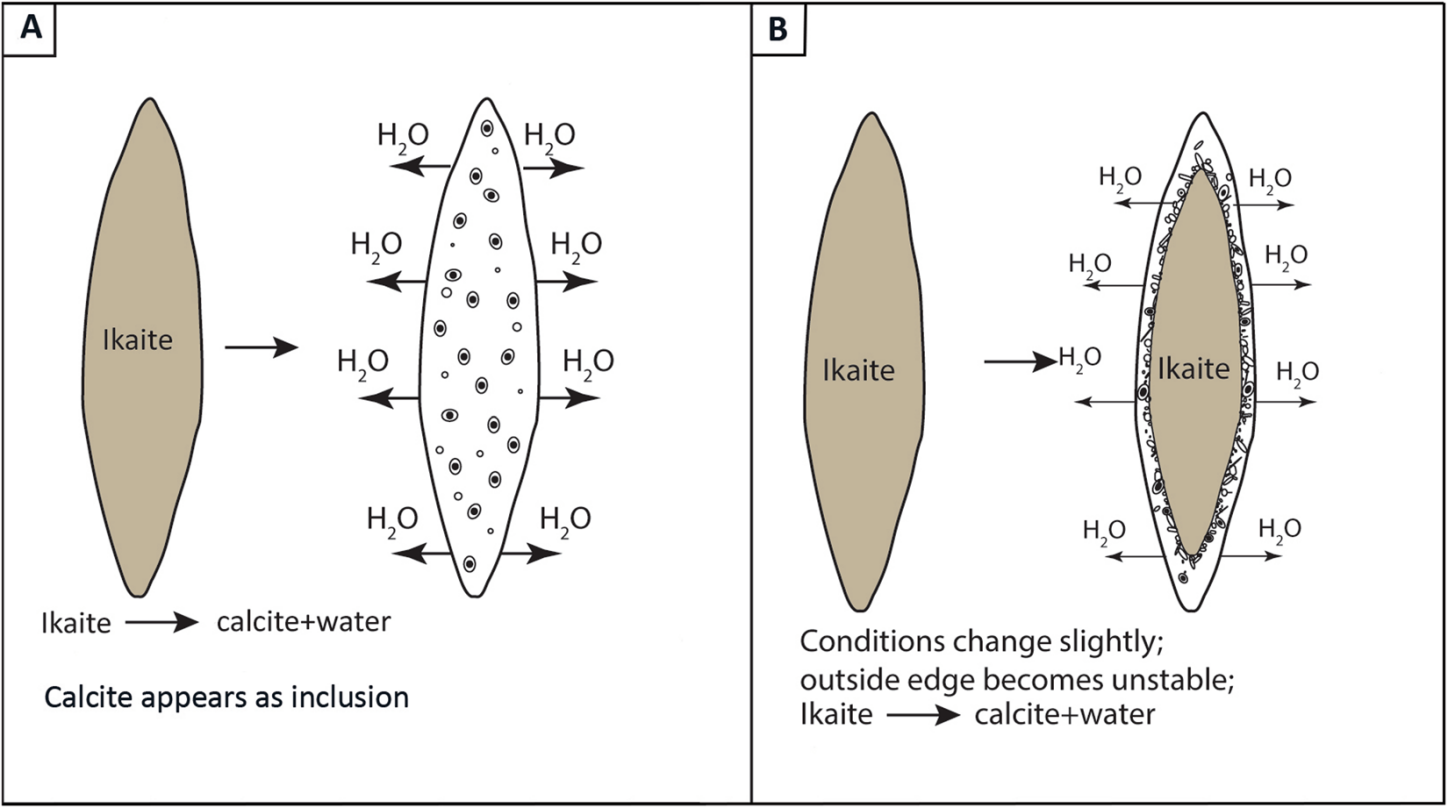
Ikaite to glendonite transformation models proposed by Vickers *et al*. 2018. (**A**) Ikaite to glendonite transformation starting with calcite inclusions. (**B**) A change in outside conditions resulting in the transformation of ikaite from the outside towards the inside of the crystal. Modified from Vickers *et al*.^49^.

We observed two distinct textures of the ikaite crystals in the transition zone. The first type of texture (Figs. 3, 4 and 6B) is the perfect pseudomorphic replacement of ikaite by porous calcite. The second texture was seen in two samples (7b and 11) from the transition zone (Figs. 2 and 7). The ikaite crystals have rounded cavities, which contain undefined material and small crystals with calcite shape (Fig. 7B). Sample 7b contains both intact ikaite crystals and ikaite with cavities (Figs. 2A and 7B). Sample 11 contains ikaite crystals and ikaite with cavities but also pseudomorphs after ikaite (Figs. 2B,C, 7A). Vickers *et al*. (2018) proposed 2 models for the ikaite to glendonite transformation, based on observations and data from their study and previous studies on glendonite^49^ (Fig. 8). The model in Fig. 8A was suggested for rapid change in conditions and the model in Fig. 8B for no change or small change in conditions^49^. The model in Fig. 8A corresponds to exps. 7b and 11 (Figs. 2 and 7), however there were no abrupt change in the conditions during our experiments that could explain this change. The model in Fig. 8B corresponds to exps. 5, 6b, 7a, 8a, 8b, 11 and 18 (Figs. 3, 4 and 6B) and indeed our results indicate that a slight change in conditions (e.g. pH and/or temperature) induced this transformation. Sanchez-Pastor *et al*. (2016) made similar observation when they exposed ikaite crystals to air at 10 °C and at 20 °C. Ikaite crystals exposed at 10 °C were pseudomorphically replaced, whereas crystals exposed at 20 °C recrystallized to calcite and vaterite^50^. It could be that an abrupt change in temperature or other conditions would provoke partial dissolution of ikaite and crystallization of calcite or other phases, whereas a slight change in conditions favours pseudomorphic transformation.

### Controls on Mg-rich Amorphous Calcium Carbonate (ACC) formation

In our study we focused on ikaite nucleation, however we made some interesting observations on Mg-rich ACC formation that are worth discussing. With only one exception, we did not detect ACC below pH 10.31 in the parent solution (Table 2), even though ACC had a slightly higher SI than ikaite in all series at 35 °C (Supplementary Fig. S3). However, it is possible that the SI for Mg rich ACC differs from pure ACC.

In series 5 (pH 10.31) ikaite precipitated together with Mg rich ACC at temperatures ≥10 °C and the amount of ACC increased with temperature. Blue and Dove (2015) demonstrated from experimental studies on ACC that the incorporation of Mg into ACC was controlled by Mg/Ca ratio and pH^51^. For experiments at pH 9.5 to 10.3 the Mg content in ACC in their study was 31–65% which is in agreement with our result of 35 ± 10%. It has also been found that the incorporation of Mg into ACC stabilises this otherwise unstable phase^52,53^. This would explain why we did not observe any transformation of ACC in our samples. In the experiments at pH 10.31 > 5 °C ikaite and ACC co-precipitate and both phases have similar predicted SI (Supplementary Fig. S3). However, ACC is favoured by the increase in temperature, whereas the amount of ikaite diminished (Table 2).

## Conclusions

Pseudomorphs (glendonites) after ikaite are widely used as paleotemperature indicators because ikaite has only been observed in nature at temperatures below 7 °C. The results from our study show that ikaite can nucleate at temperatures up to 35 °C for pH ranging from 9.3 to 10.3. Based on geochemical modelling, we infer that the concentration of the CaCO_3_^0^ ion pair in the parent solution is an important control of ikaite precipitation.

Even though ikaite crystals precipitated at these higher temperatures do not survive, their pseudomorphs do, and can be misinterpreted as indicator of low paleo temperatures. Pseudomorphic replacement of ikaite by calcite can occur rapidly (<5 hours) in response to minor changes of pH and/or temperature. We therefore challenge the use of glendonite as a paleotemperature indicator.

Finally, the broad temperature range (≤35 °C) for ikaite nucleation would suggest that ikaite could be a common transition mineral that competes with ACC formation in marine environments. ACC can incorporate Mg (<65%) whereas ikaite only contain traces of Mg. It is therefore possible that preserved low-Mg calcite commonly derives from ikaite in marine environments, whereas Mg-rich ACC is the major precursor of Mg-rich calcite.

## Supplementary information

Supplementary Information.

## References

[1] Berner RA (1975). The role of magnesium in the crystal growth of calcite and aragonite from sea water. Geochimica et Cosmochimica Acta.

[2] Dove PM, Hochella MF (1993). Calcite precipitation mechanisms and inhibition by orthophosphate: *In situ* observations by Scanning Force Microscopy. Geochimica et cosmochimica acta.

[3] Katz JL, Reick MR, Herzog RE, Parsiegla KI (1993). Calcite growth inhibition by iron. Langmuir.

[4] Stanley SM, Hardie LA (1998). Secular oscillations in the carbonate mineralogy of reef-building and sediment-producing organisms driven by tectonically forced shifts in seawater chemistry. Palaeogeography, Palaeoclimatology, Palaeoecology.

[5] Ricketts RD, Johnson TC, Brown ET, Rasmussen KA, Romanovsky VV (2001). The Holocene paleolimnology of Lake Issyk-Kul, Kyrgyzstan: Trace element and stable isotope composition of ostracodes. Palaeogeography, Palaeoclimatology, Palaeoecology.

[6] Taylor GF (1975). The occurrence of monohydrocalcite in two small lakes in the south-east of South Australia. American Mineralogist: Journal of Earth and Planetary Materials.

[7] Swainson IP (2008). The structure of monohydrocalcite and the phase composition of the beachrock deposits of Lake Butler and Lake Fellmongery, South Australia. American Mineralogist.

[8] Han Z (2018). The Significant Roles of Mg/Ca Ratio, Cl− and SO42− in Carbonate Mineral Precipitation by the Halophile Staphylococcus epidermis Y2. Minerals.

[9] Pauly H (1963). “Ikaite”, A New Mineral from Greenland. Arctic v.

[10] Buchardt B, Israelson C, Seaman P, Stockmann G (2001). Ikaite tufa in Ikka Fjord, southwest Greenland: Their formation by mixing of seawater and alkaline spring water. Journal of Sedimentary Research, v.

[11] Huggett JM, Schultz BP, Shearman DJ, Smith AJ (2005). The petrology of ikaite pseudomorphs and their diagenesis. Proceedings of the Geologists’ Association.

[12] Suess E (1982). Calcium carbonate hexahydrate from organic-rich sediments of the antarctic shelf: precursors of glendonites. Science.

[13] Kodina, L. A., Tokarev, L. N., Vlasova, L. N. & Korobeinik, G. S. Contribution of biogenic methane to ikaite formation in the Kara Sea. In: Stein, R., Fahl, K., Futterer, D., Galimov, E. M., Stepanets, O. V. (Eds.), Siberian river run-off in the environmental significance. Elsevier, Amsterdam, Boston, Heidelberg, London, New York, pp. 349–375 (2003).

[14] Lu Z (2012). An ikaite record of late Holocene climate at the Antarctic Peninsula. Earth and Planetary Science Letters.

[15] Dieckmann GS (2008). Calcium carbonate as ikaite crystals in Antarctic sea ice. Geophysical Research Letters.

[16] Dieckmann G (2010). Ikaite (CaCO_3_. 6H_2_O) discovered in Arctic sea ice. The Cryosphere.

[17] Field LP (2017). Unusual morphologies and the occurrence of pseudomorphs after ikaite (CaCO_3_ 6H_2_O) in fast growing, hyperalkaline speleothems. Mineralogical Magazine.

[18] Council TC, Bennett PC (1993). Geochemistry of ikaite formation at Mono Lake, California: implications for the origin of tufa mounds. Geology, v.

[19] Oehlerich MB (2009). On the study of natural and synthetic ikaite crystals. Revista de la Sociedad Española de Mineralogía.

[20] Ito T (1996). Ikaite from cold spring water at Shiowakka, Hokkaido, Japan. J. Mm. Petr. Econ. Geol..

[21] Boch R (2015). Rapid ikaite (CaCO_3_·6H_2_O) crystallization in a man-made river bed: hydrogeochemical monitoring of a rarely documented mineral formation. Applied geochemistry.

[22] Swainson IP, Hammond RP (2001). Ikaite, CaCO_3_ 6H_2_O: Cold comfort for glendonites as paleothermometers. American Mineralogist.

[23] Frank TD, Thomas SG, Fielding CR (2008). On using carbon and oxygen isotope data from glendonites as paleoenvironmental proxies: a case study from the Permian system of eastern Australia. Journal of Sedimentary Research.

[24] McLachlan IR, Tsikos H, Cairncross B (2001). Glendonites (pseudomorphs after ikaite) in late carboniferous Marine Dwyka beds in Southern Africa. South African Journal of Geology.

[25] Rogov, M. A. & Zakharov, V. A. Jurassic and Lower Cretaceous glendonite occurrences and their implication for Arctic paleoclimate reconstructions and stratigraphy. Earth Science Frontiers, 17(Special issue), 345–346 (2010).

[26] Vickers, M. L. *et al* The duration and magnitude of Cretaceous cool events: Evidence from the northern high latitudes. *Geological Society of America Bulletin* (2019)..

[27] Teichert BM, Luppold FW (2009). Glendonite formation in Early Jurassic dark shales–evidence for methane seepage in northern Germany. Geochimica et Cosmochimica Acta.

[28] Selleck BW, Carr PF, Jones BG (2007). A review and synthesis of glendonites (pseudomorphs after ikaite) with new data: assessing applicability as recorders of ancient coldwater conditions. Journal of Sedimentary Research.

[29] Rickaby REM (2006). Potential of ikaite to record the evolution of oceanic δ18O. Geology.

[30] Dempster T, Jess SA (2015). Ikaite pseudomorphs in Neoproterozoic Dalradian slates record Earth’s coldest metamorphism. Journal of the Geological Society.

[31] Bischoff JL, Fitzpatrick JA, Rosenbauer RJ (1993). The solubility and stabilization of ikaite (CaCO3· 6H2O) from 0 to 25 C: Environmental and paleoclimatic implications for thinolite tufa. The. Journal of Geology.

[32] Hu YB, Wolf-Gladrow DA, Dieckmann GS, Völker C, Nehrke G (2014). A laboratory study of ikaite (CaCO3· 6H2O) precipitation as a function of pH, salinity, temperature and phosphate concentration. Marine Chemistry.

[33] Tollefsen E (2018). Chemical controls on ikaite formation. Mineralogical Magazine.

[34] Clarkson JR, Price TJ, Adams CJ (1992). Role of metastable phases in the spontaneous precipitation of calcium carbonate. Journal of the Chemical Society, Faraday Transactions.

[35] Stockmann G (2018). Control of a calcite inhibitor (phosphate) and temperature on ikaite precipitation in Ikka Fjord, southwest Greenland. Applied Geochemistry.

[36] Popov LE (2019). Glendonite occurrences in the Tremadocian of Baltica: first Early Palaeozoic evidence of massive ikaite precipitation at temperate latitudes. Scientific reports.

[37] Parkhurst, D. L. & Appelo, C. A. J. Description of input and examples for PHREEQC version 3: a computer program for speciation, batch-reaction, one-dimensional transport, and inverse geochemical calculations (No. 6-A43). US Geological Survey (2013).

[38] Plummer LN, Busenberg E (1982). The solubilities of calcite, aragonite and vaterite in CO_2_-H_2_O solutions between 0 and 90 C, and an evaluation of the aqueous model for the system CaCO_3_-CO_2_-H_2_O. Geochimica et cosmochimica acta.

[39] Brecevic L, Nielsen AE (1989). Solubility of amorphous calcium carbonate. Journal of Crystal Growth.

[40] Busenberg E, Plummer LN (1989). Thermodynamics of magnesian calcite solid-solutions at 25 C and 1 atm total pressure. Geochimica et Cosmochimica Acta.

[41] Gal JY, Bollinger JC, Tolosa H, Gache N (1996). Calcium carbonate solubility: a reappraisal of scale formation and inhibition. Talanta.

[42] Genovese D (2016). Role of CaCO3 neutral pair in calcium carbonate crystallization. Crystal growth & design.

[43] Lennie AR, Tang CC, Thompson SP (2004). The structure and thermal expansion behaviour of ikaite, CaCO_3_· 6H_2_O, from T= 114 to T= 293 K. Mineralogical Magazine.

[44] Tang CC (2009). The ikaite-to-vaterite transformation: new evidence from diffraction and imaging. Journal of Applied Crystallography.

[45] Tateno, N. & Kyono, A. Structural change induced by dehydration in ikaite (CaCO3 6H2O). *Journal of Mineralogical and Petrological Sciences*, 140320 (2014).

[46] Teichert BMA, Luppold FW (2013). Glendonites from an Early Jurassic methane seep—Climate or methane indicators?. Palaeogeography, Palaeoclimatology, Palaeoecology.

[47] Putnis A, Putnis CV (2007). The mechanism of reequilibration of solids in the presence of a fluid phase. Journal of Solid State Chemistry.

[48] Hellmann R (2015). Nanometre-scale evidence for interfacial dissolution–reprecipitation control of silicate glass corrosion. Nature materials.

[49] Vickers M, Watkinson M, Price GD, Jerrett R (2018). An improved model for the ikaite-glendonite transformation: evidence from the Lower Cretaceous of Spitsbergen, Svalbard. Norwegian Journal of Geology.

[50] Sánchez-Pastor N (2016). Crystallization of ikaite and its pseudomorphic transformation into calcite: Raman spectroscopy evidence. Geochimica et Cosmochimica Acta.

[51] Blue CR, Dove PM (2015). Chemical controls on the magnesium content of amorphous calcium carbonate. Geochimica et Cosmochimica Acta.

[52] Lin CJ, Yang SY, Huang SJ, Chan JC (2015). Structural characterization of Mg-stabilized amorphous calcium carbonate by Mg-25 solid-state NMR spectroscopy. *The*. Journal of Physical Chemistry C.

[53] Konrad F (2018). Influence of aqueous Mg concentration on the transformation of amorphous calcium carbonate. Journal of Crystal Growth.

