# Supplementary material for: Ikaite nucleation at 35 °C challenges the use of glendonite as a paleotemperature indicator
Source: Sci Rep. 2020 May 18;10:8141. doi: 10.1038/s41598-020-64751-5 (PMC7235076; doi:10.1038/s41598-020-64751-5)
Supplement: Supplementary file 1 — Supplementary Information. [file 41598_2020_64751_MOESM1_ESM.pdf]

Supplementary Information

Ikaite nucleation at 35°C challenges the use of glendonite as a paleotemperature indicator

Elin Tollefsen[1,*], Tonci Balic-Zunic[2], Carl-Magnus Mörth[1], Volker Bruchert[1], Cheng Choo Lee[3] and Alasdair Skelton[1]

[1]Department of Geological Sciences, Stockholm University, 106 91 Stockholm, Sweden, *elin.tollefsen@geo.su.se, [2]Department of Geosciences and Natural Resource Management, University of Copenhagen, 1350 Copenhagen, Denmark, [3]Chemical Biological Centre, Umeå University, 901 87 Umeå Sweden

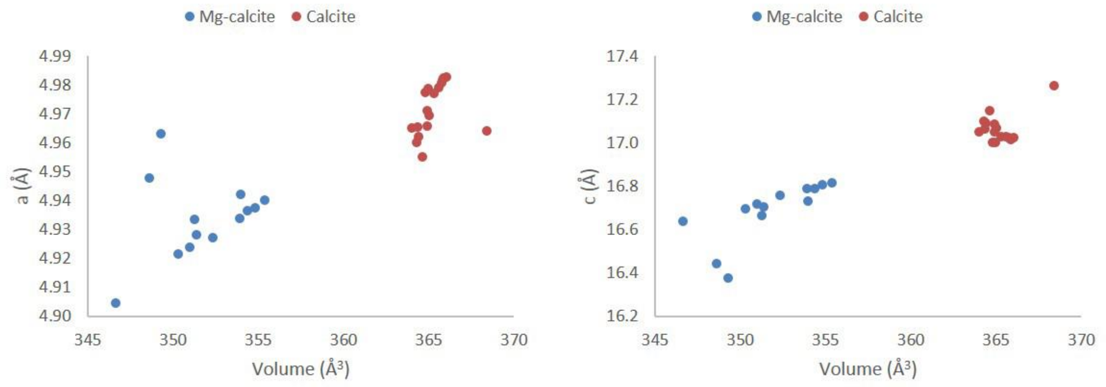

Figure S1. Unit cell data for Mg-calcite and calcite.

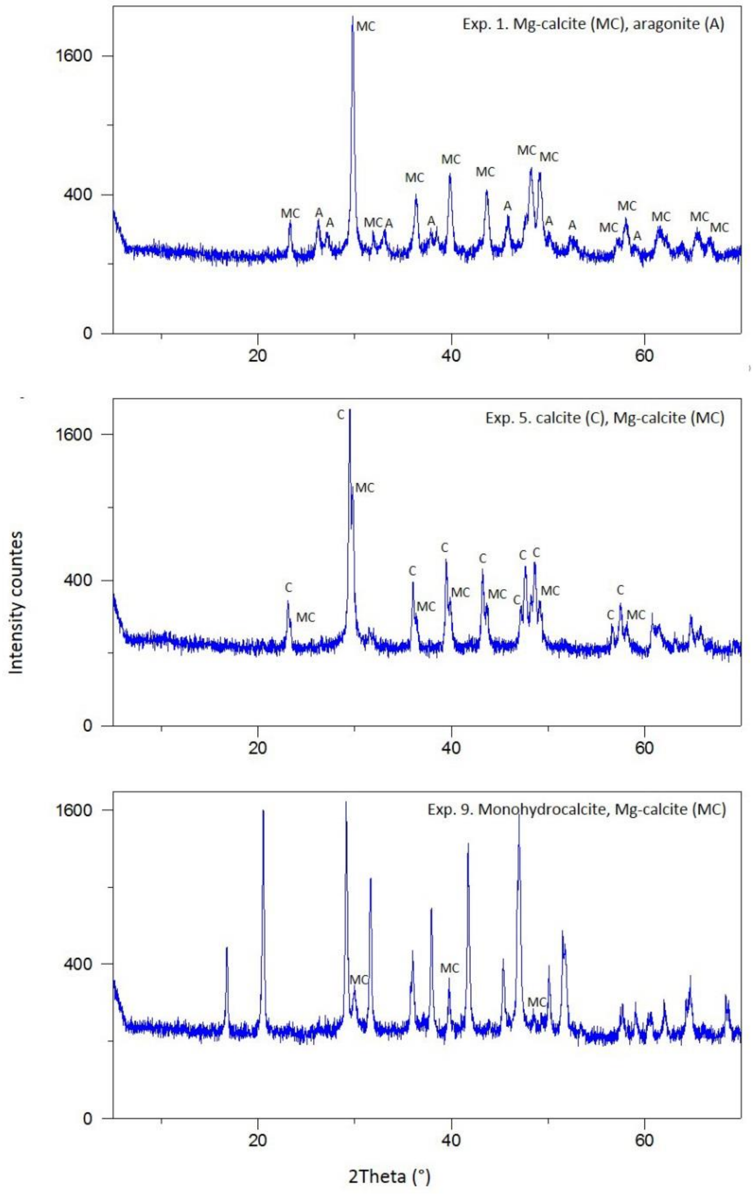

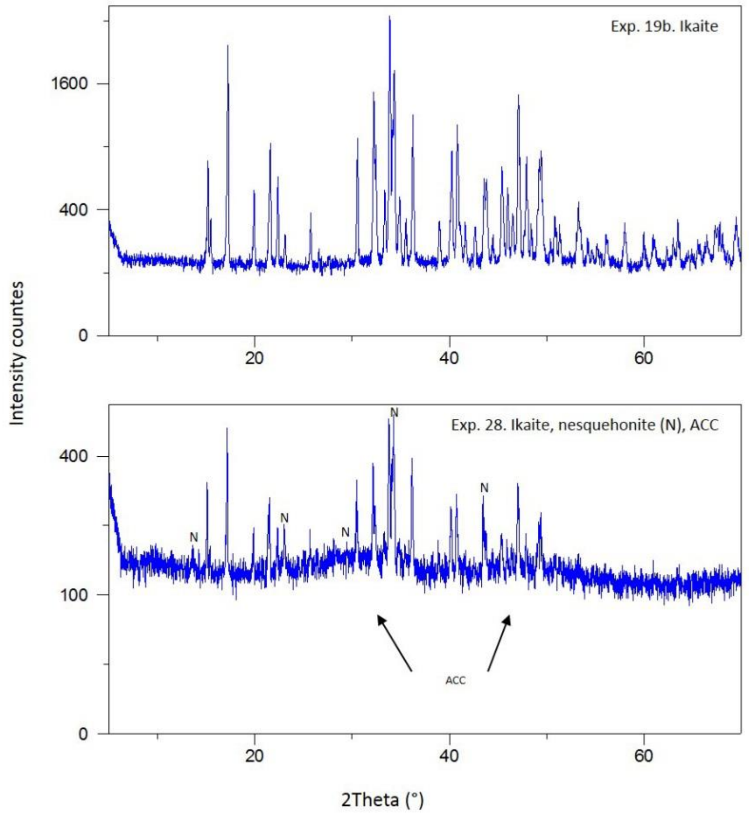

Figure S2. X-ray powder diffraction spectra for exp. 1, 5, 9, 19b and 28. Exp. 1 shows peaks for Mg-calcite (MC) and aragonite (A). Exp. 5 shows peaks for calcite (C) and Mg-calcite (MC). Exp. 9 shows peaks for monohydrocalcite (unlabelled) and Mg-calcite (MC). Exp. 19b shows peaks for only ikaite. Exp. 28 shows peaks for ikaite (unlabelled), nesquehonite (N) and amorphous calcium carbonate (ACC) indicated by the arrows.

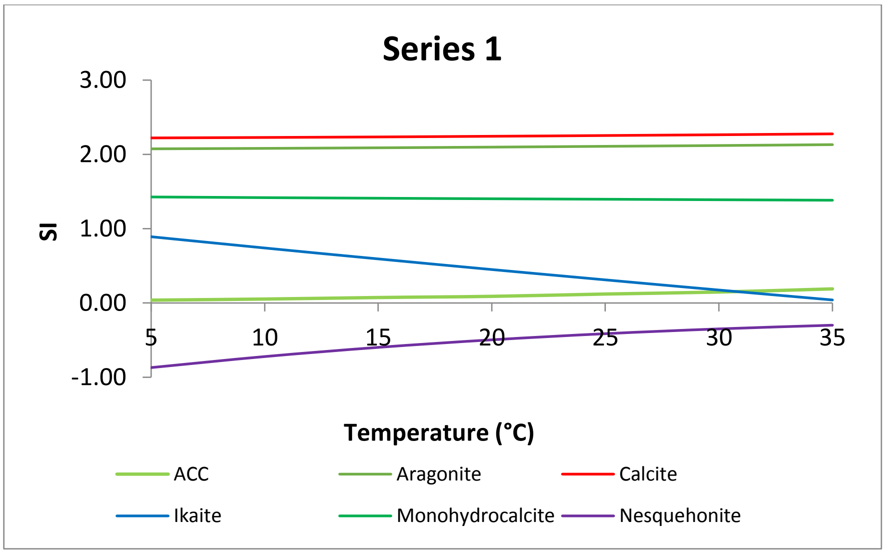

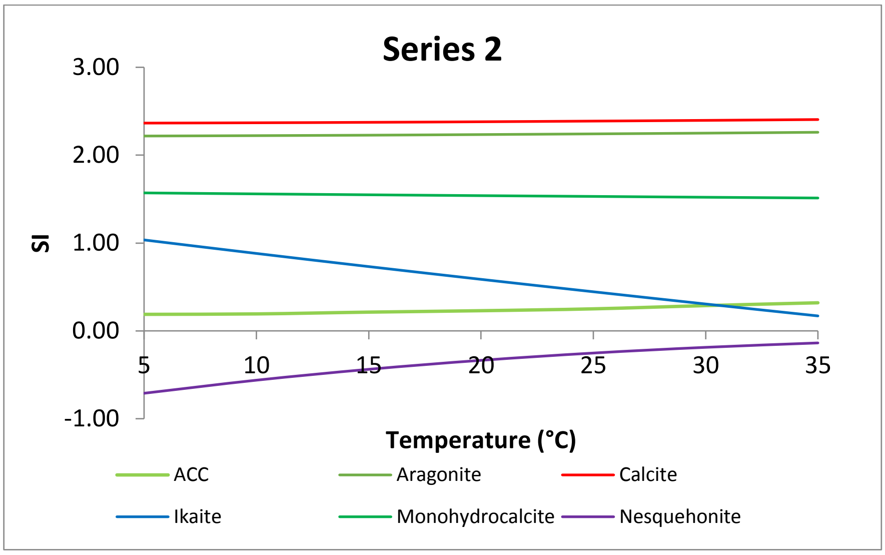
Series 2
SI
3.00
2.00
1.00
0.00
-1.00
5
10
15
20
25
30
35
Temperature (°C)
ACC
Aragonite
Calcite
Ikaite
Monohydrocalcite
Nesquehonite

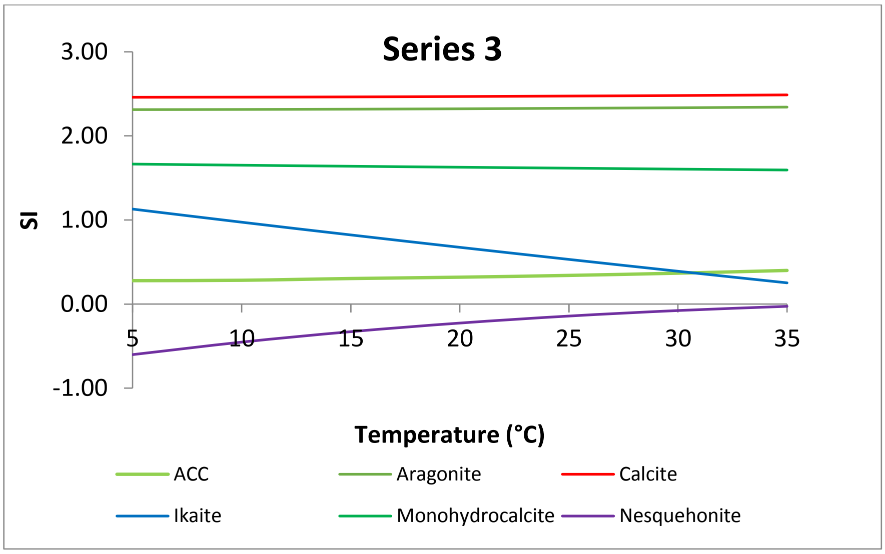
Series 3
SI
3.00
2.00
1.00
0.00
-1.00
5
10
15
20
25
30
35
Temperature (°C)
ACC
Aragonite
Calcite
Ikaite
Monohydrocalcite
Nesquehonite

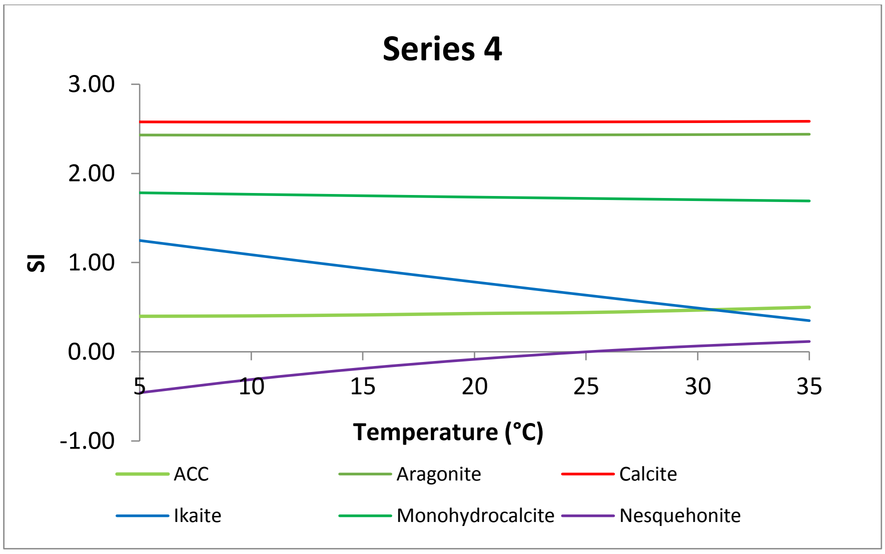
Series 4
SI
3.00
2.00
1.00
0.00
-1.00
5
10
15
20
25
30
35
Temperature (°C)
ACC
Aragonite
Calcite
Ikaite
Monohydrocalcite
Nesquehonite

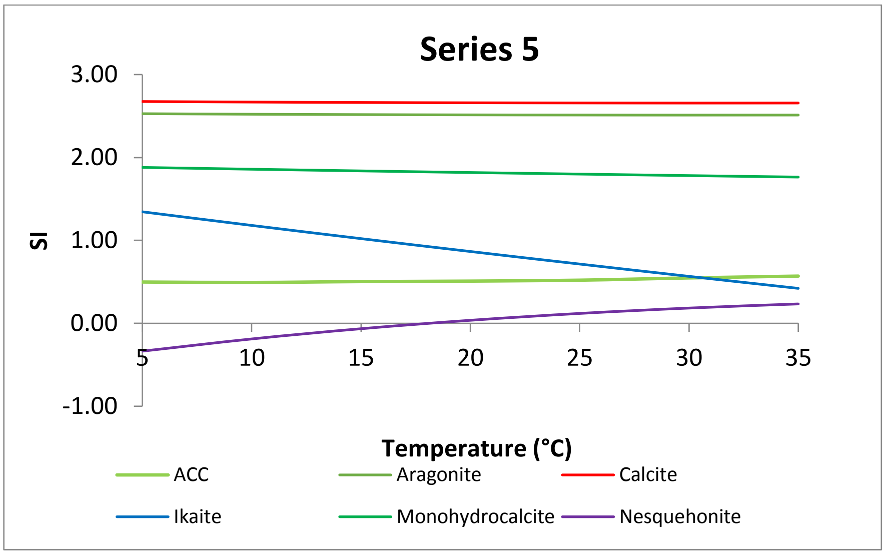

Figure S3. Saturation indices (SI) calculated with PhreeqC.

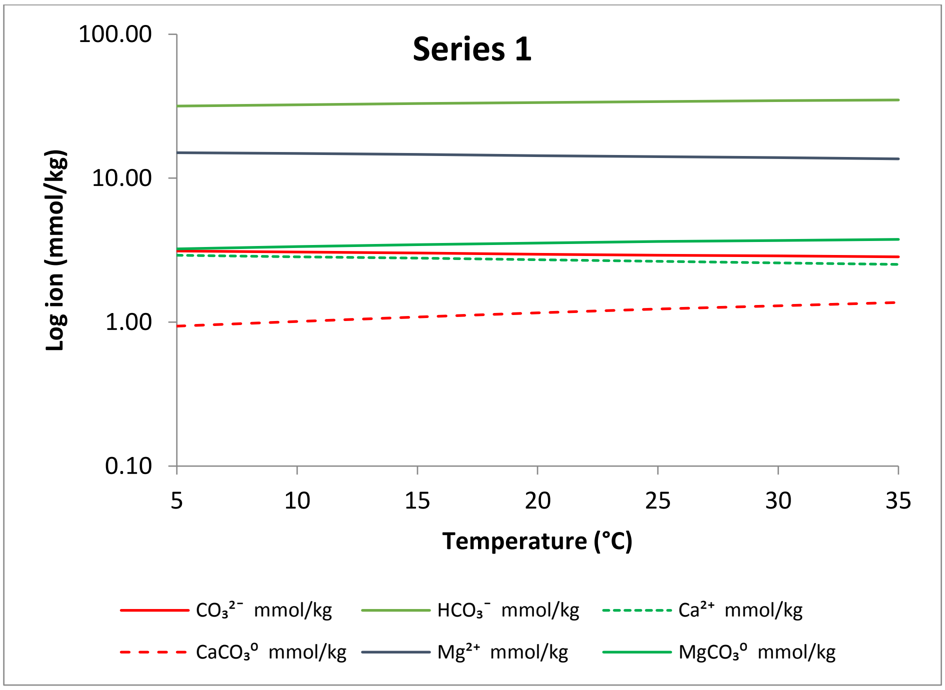

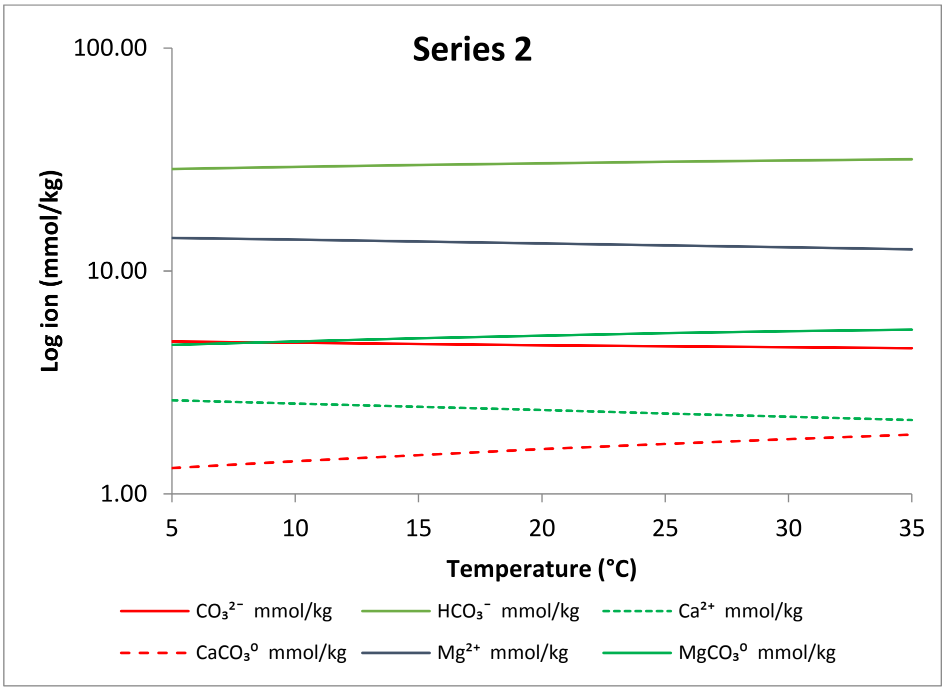
Series 2
100.00
10.00
1.00
Log ion (mmol/kg)
5
10
15
20
25
30
35
Temperature (°C)
CO₃²⁻ mmol/kg
HCO₃⁻ mmol/kg
Ca²⁺ mmol/kg
CaCO₃⁰ mmol/kg
Mg²⁺ mmol/kg
MgCO₃⁰ mmol/kg

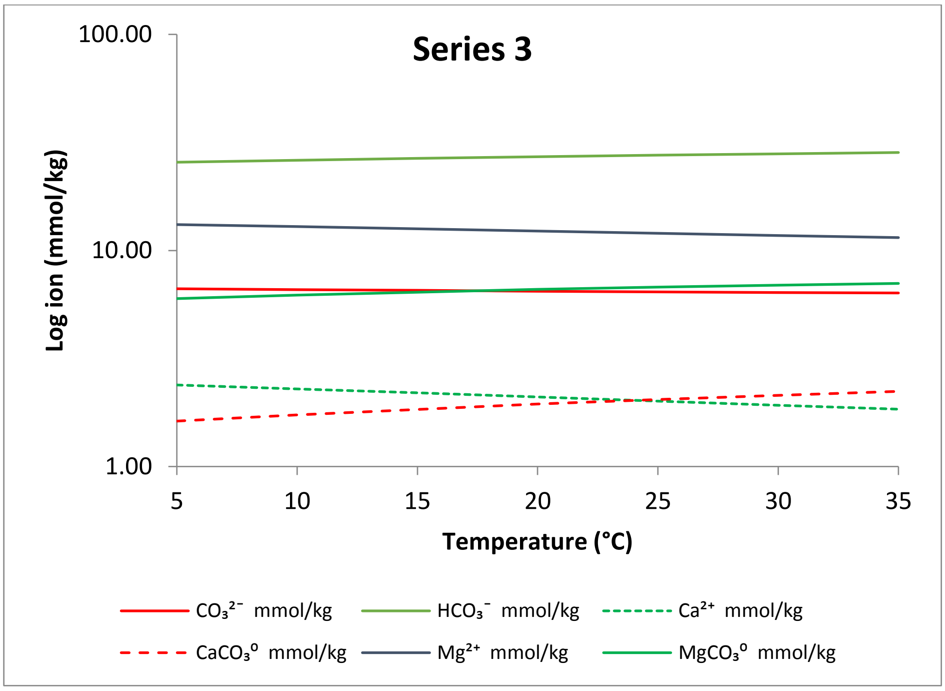
Series 3
100.00
10.00
1.00
Log ion (mmol/kg)
5
10
15
20
25
30
35
Temperature (°C)
CO₃²⁻ mmol/kg
HCO₃⁻ mmol/kg
Ca²⁺ mmol/kg
CaCO₃⁰ mmol/kg
Mg²⁺ mmol/kg
MgCO₃⁰ mmol/kg

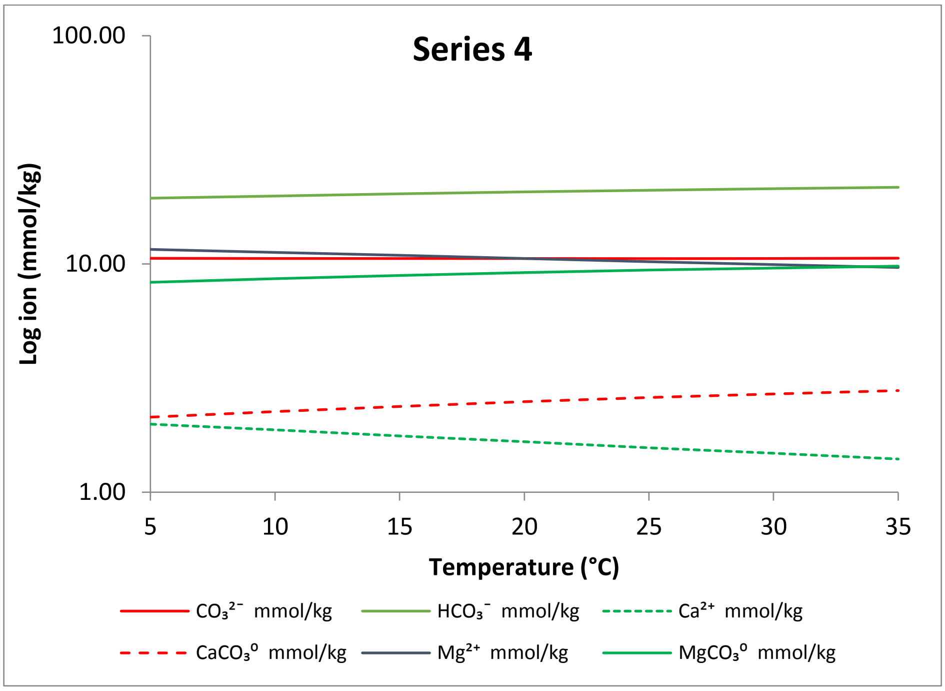

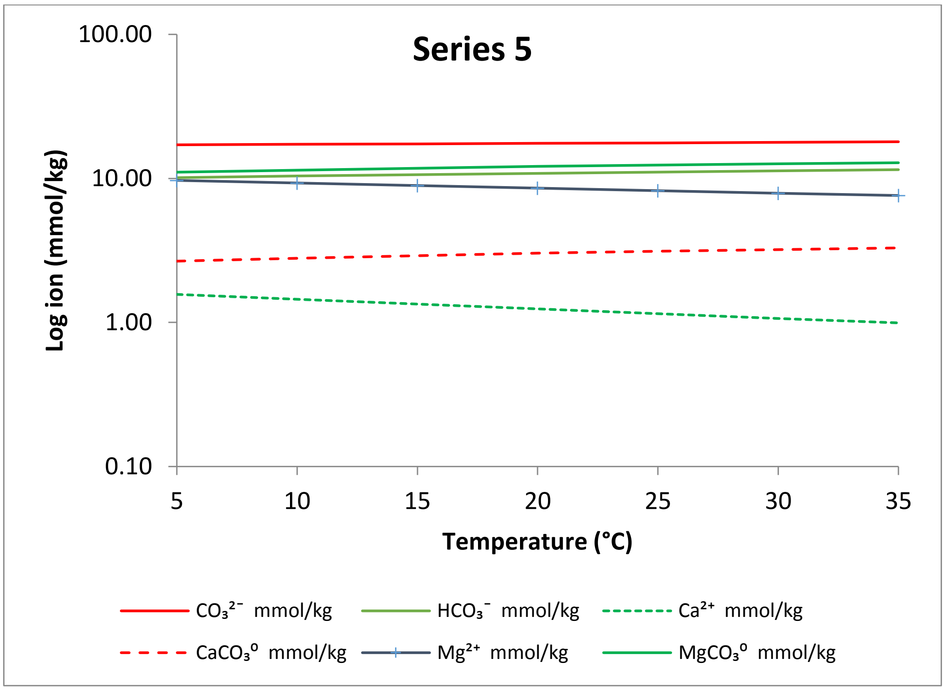

Figure S4. Ion concentrations calculated with PhreeqC.

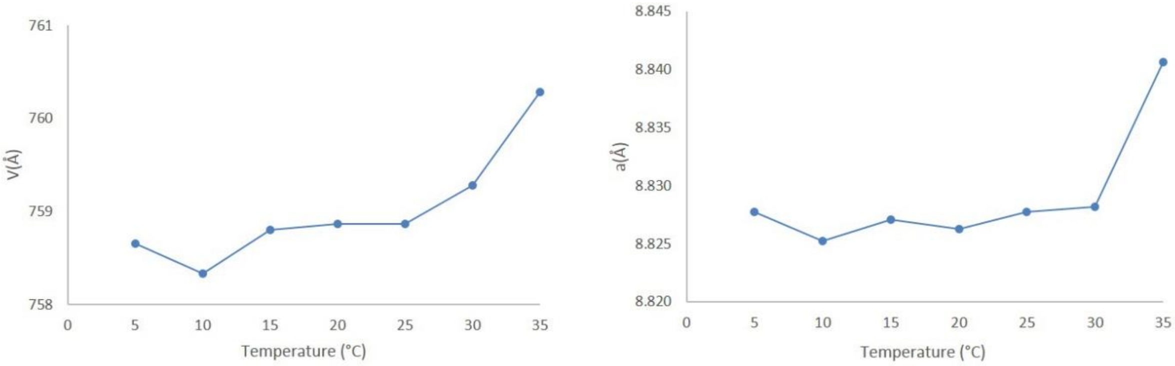

Figure S5. Variation in lattice parameters (Volume and a-axis) for ikaite with temperature in Series 4.

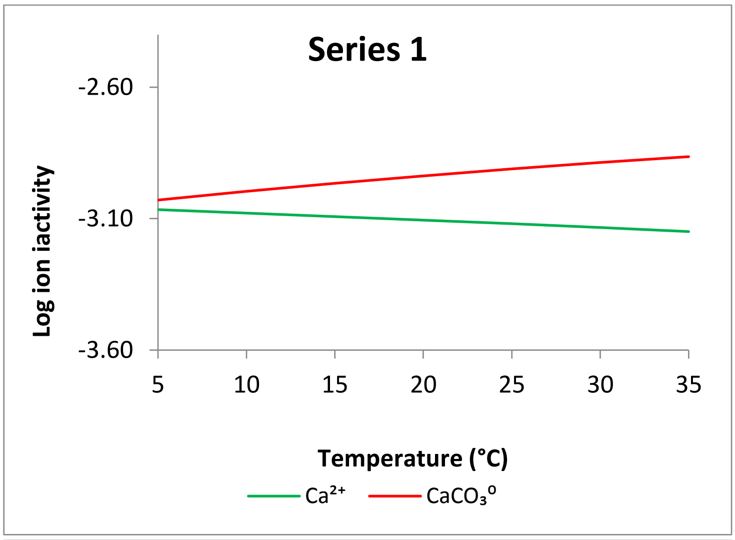

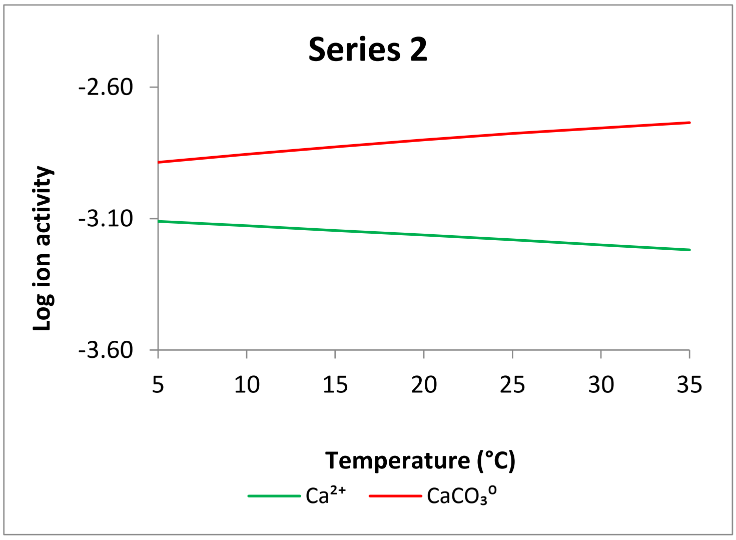

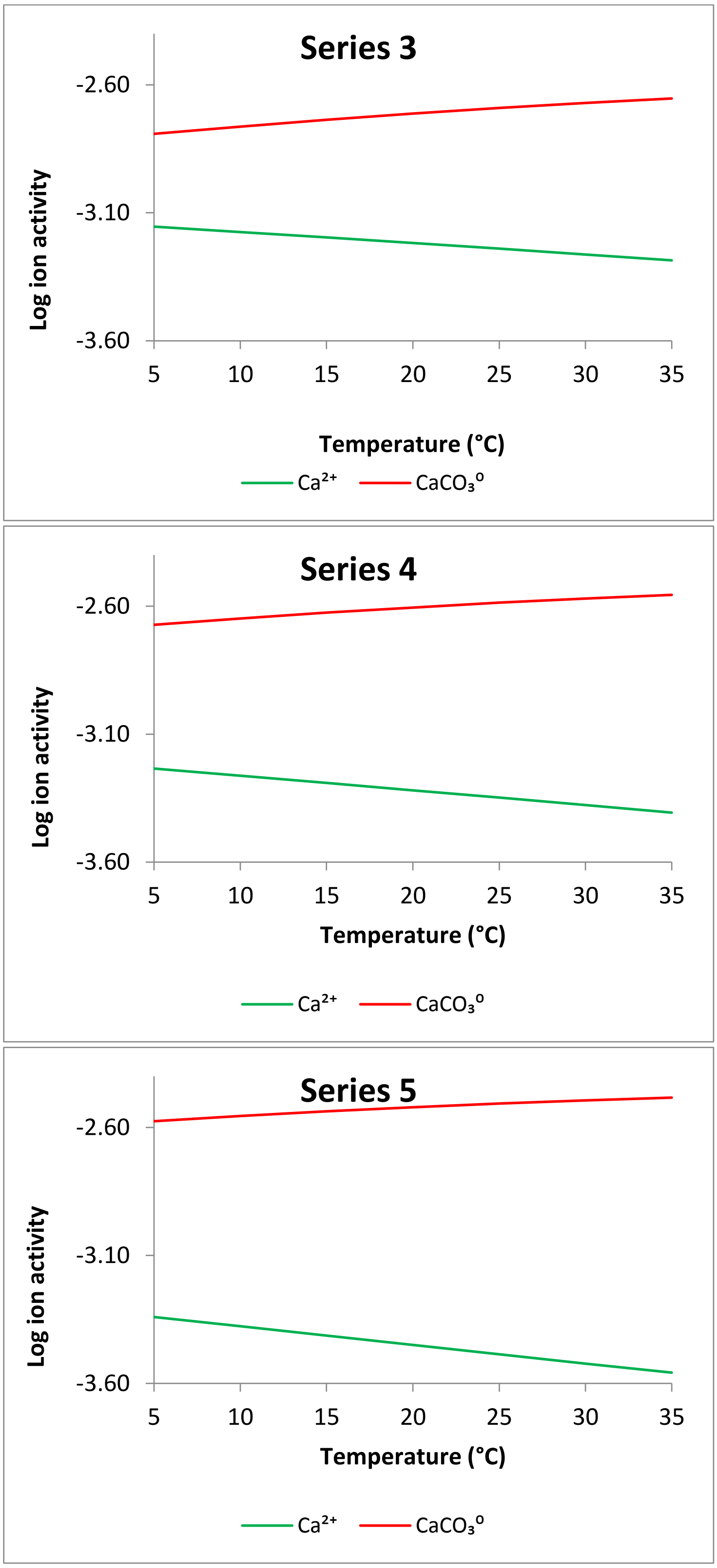

Figure S6. Log ion activity calculated with PhreeqC.
